# The A-Current Modulates Learning via NMDA Receptors Containing the NR2B Subunit

**DOI:** 10.1371/journal.pone.0024915

**Published:** 2011-09-26

**Authors:** Ángela Fontán-Lozano, Irene Suárez-Pereira, David González-Forero, Ángel Manuel Carrión

**Affiliations:** 1 División de Neurociencias, Universidad Pablo de Olavide de Sevilla, Sevilla, Spain; 2 Área de Fisiología, Facultad de Medicina, Universidad de Cádiz, Cádiz, Spain; University of North Dakota, United States of America

## Abstract

Synaptic plasticity involves short- and long-term events, although the molecular mechanisms that underlie these processes are not fully understood. The transient A-type K^+^ current (I_A_) controls the excitability of the dendrites from CA1 pyramidal neurons by regulating the back-propagation of action potentials and shaping synaptic input. Here, we have studied how decreases in I_A_ affect cognitive processes and synaptic plasticity. Using wild-type mice treated with 4-AP, an I_A_ inhibitor, and mice lacking the DREAM protein, a transcriptional repressor and modulator of the I_A_, we demonstrate that impairment of I_A_ decreases the stimulation threshold for learning and the induction of early-LTP. Hippocampal electrical recordings in both models revealed alterations in basal electrical oscillatory properties toward low-theta frequencies. In addition, we demonstrated that the facilitated learning induced by decreased I_A_ requires the activation of NMDA receptors containing the NR2B subunit. Together, these findings point to a balance between the I_A_ and the activity of NR2B-containing NMDA receptors in the regulation of learning.

## Introduction

Memory and synaptic plasticity are mediated by two distinct components. The first yields only transient phenomena, short-term memory (STM, lasting minutes to hours) and the early phase of LTP (E-LTP, lasting 0.5–1 hr), while the second involves synaptic changes and the activation of mechanisms that stabilize the memory, resulting in long-term memory (LTM, lasting days, weeks or years), and the late phase of LTP (L-LTP, lasting many hours). Distinct molecular mechanisms are thought to underlie each component [1] since modifications of pre-existing proteins are sufficient for the transient changes, while new gene expression (transcription and translation) is required for sustained changes [1]–[7].

Kv4 channels are the main contributors to the potassium A-current (I_A_) [8]. These channels are concentrated somato-dendritically, where they act as crucial regulators of postsynaptic excitability [9]–[11] and modulators of synaptic plasticity [12]–[17]. DREAM is a multifunctional Ca^2+^-binding protein of the EF-hand subfamily of neuronal calcium sensors and it displays specific roles in different cell compartments [18]–[19]. In the nucleus, DREAM acts as a Ca^2+^-dependent transcriptional repressor, regulating gene expression [18], [20]–[23], while outside the nucleus, it interacts with Kv4 potassium channels, directing their trafficking to the plasma membrane and regulating channel gating properties [19], [24]. In addition, DREAM appears to directly or indirectly affect synaptic plasticity by modulating NMDA receptors (NMDARs) [25]–[26]. Furthermore, NMDAR, especially the NR2B subunit, and Kv4.2 channels activity are mutually regulated to modulate synaptic plasticity [27]–[29].

We recently described the role of DREAM, acting as transcriptional repressor, in the regulation of learning and memory [30]. In the present study, we have used pharmacological (I_A_ inhibition with 4-AP) and genetic (*dream^−/−^* mice) approaches to investigate the role of I_A_ in synaptic plasticity, and in learning and memory. Both models showed similar alterations in basal electrical hippocampal activity, as well as a decrease in the stimulation threshold for learning and early-LTP induction. We also demonstrate that the facilitation of learning induced by I_A_ inhibition is mediated by NMDARs containing the NR2B subunit. These results suggest that, via an inhibitory modulation of NR2B-containing NMDARs, the I_A_ determines the stimulation threshold and consequently dictates how quickly the learning process occurs.

## Results

### The transient outward A-type potassium current (I_A_) is strongly reduced in CA1 pyramidal neurons of *dream^−/−^* mice

**Figure 1:**
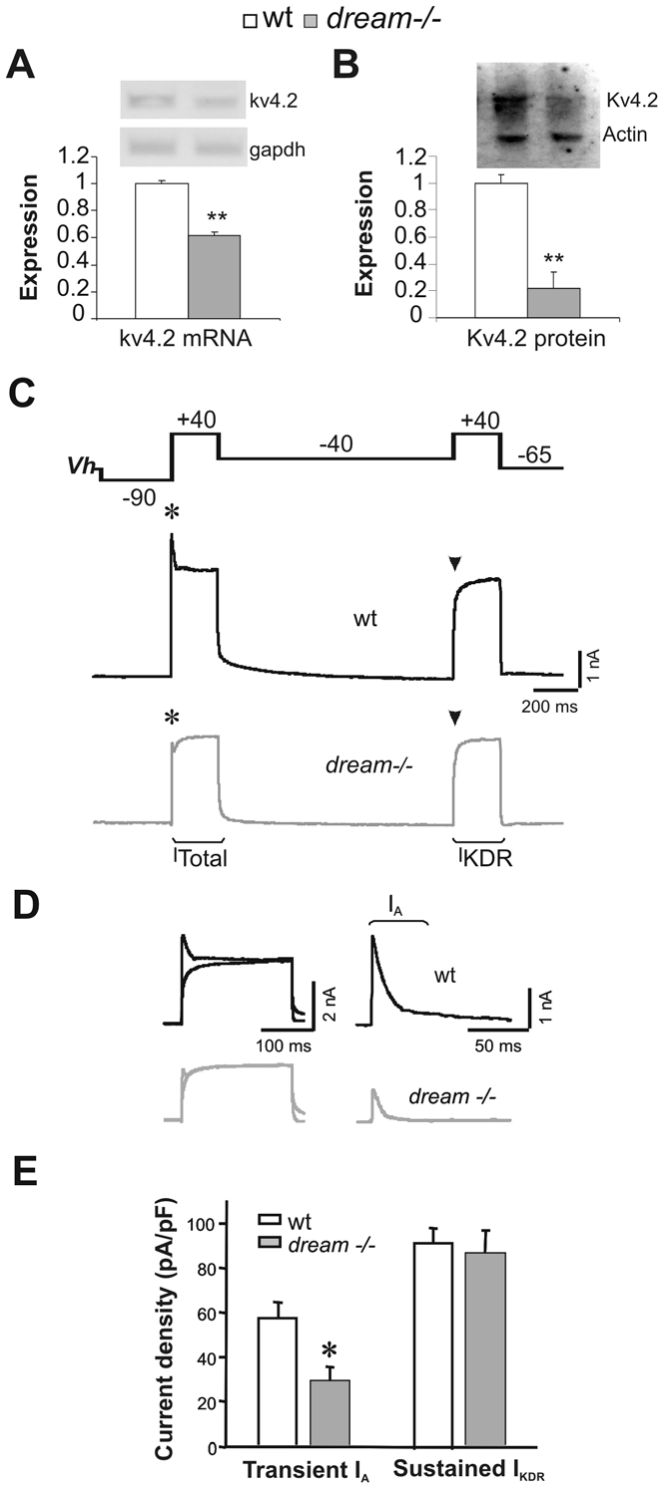
Kv4.2 expression and I_A_ activity are reduced in the hippocampus of *dream^−/−^* mice. Basal expression of kv4.2 mRNA (**A**) and Kv4.2 protein (**B**) in the hippocampus of wt (white bars) and *dream^−/−^* (gray bars) mice. *Gapdh* mRNA and actin protein served as internal controls. (**C**) The two-step voltage clamp protocol used to activate whole-cell voltage-dependent K^+^ currents, and representative examples of the outward currents in CA1 pyramidal neurons from wt and *dream^−/−^* mice. The first depolarizing step activated a mixed current (I_Total_) with a transient A-type component (I_A_; asterisks) and sustained non-inactivating K^+^ current (I_KDR_). I_A_ was inactivated during the second depolarizing pulse (arrowheads) leaving only the sustained I_KDR_. (**D**) Traces on the left show the superimposition of the sequential outward current responses (depolarizing pulse segments) illustrated in C. Traces on the right show I_A_ current traces calculated by subtracting I_KDR_ from I_Total_ for the same neurons. (**E**) Summary data comparing the peak current density (amplitude normalized to cell capacitance) of I_A_ and I_KDR_ in wt and mutant neurons (n = 30 and 23 neurons from 6 mice of each genotype, respectively). * *p*<0.05, ** *p*≤0.01.

DREAM is a cytoplasmic and nuclear protein [18], [19], with distinct functions in each of these cell compartments. DREAM regulates gene expression in the nucleus, and in the cytoplasm it influences the transport and the activity of Kv4.2 channels [24], [31]. Kv4.2 channels are the main component of the I_A_ current in the hippocampus [8], where they regulate neuronal excitability [32]. Indeed, cross-regulation of Kv4.2 and DREAM protein expression was recently described [8], [33], [34] and hence, we investigated whether the absence of dream modifies Kv4.2 expression. Expression of kv4.2 mRNA and its protein decreased significantly in the hippocampus of *dream^−/−^* compared to wild-type (wt) mice (by 39.15±1.99% and 79.35±0.09%, respectively: Fig. 1A and B).

To investigate what effect this decrease in Kv4.2 channel expression had on hippocampal I_A_ in *dream^−/−^* mice, whole-cell voltage clamp recordings of voltage-dependent K^+^ currents were obtained from the soma of CA1 pyramidal neurons in slices from *dream^−/−^* and wild-type mice. In CA1 neurons, macroscopic K^+^ currents are usually composed of a transient I_A_ component and a sustained delayed current (I_KDR_), which could be separated using a two-step subtraction protocol (Fig. 1C and D). Analysis of subtracted current records revealed a significant decrease in I_A_ in the CA1 pyramidal neurons of *dream^−/−^* (29.04±6.56 pA/pF, *n* = 23 from six different mice) when compared to wt mice (57.46±6.88 pA/pF respectively, *n* = 30 from six different mice: Fig. 1E). Furthermore, this effect appeared to be specific for the I_A_ as I_KDR_ in *dream^−/−^* neurons did not differ from that in wt neurons. Overall, our electrophysiological data indicate that deletion of *dream* specifically downregulated the I_A_ component of the voltage-dependent outward K^+^ currents in CA1 pyramidal neurons.

### Diminished I_A_ facilitates learning in the object recognition test

**Figure 2:**
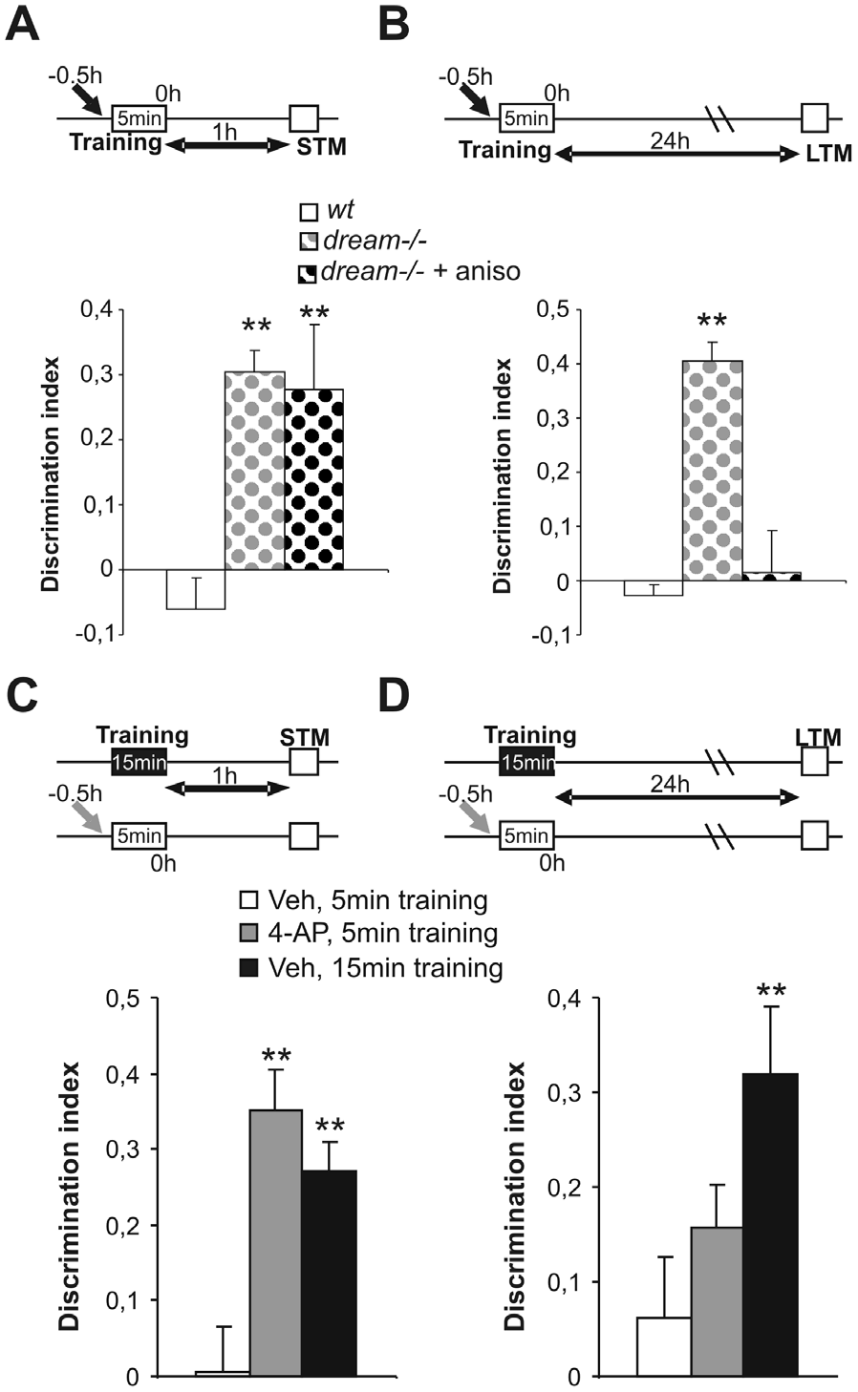
The reduction of I_A_ facilitates learning in the object recognition test. (**A**, **B**) The object recognition memory test was performed using a 5 min training session in wt (white bars) and *dream^−/−^* (gray bars) mice. Discrimination indices during short-term memory (STM, **A**) and long-term memory (LTM, **B**) sessions (1 and 24 h after training, respectively) are shown. Administration of anisomycin (aniso, black pointed bars) before the training sessions blocked the facilitation of LTM in *dream^−/−^* mice. (**C**, **D**) Administration of 4-AP before the training sessions facilitated short-term memory (STM) in wt mice. Discrimination indices during STM (**C**), and LTM (**D**) sessions are shown. n = at least 8 per group. ** *p*<0.01.

*dream^−/−^* mice display enhanced synaptic plasticity and memory consolidation, which has been linked to CREB-dependent mechanisms [30]. However, the basis for this enhanced learning remains to be fully elucidated. To study the role of new protein synthesis in enhanced learning consolidation in *dream^−/−^* mice, we administered anisomycin, an inhibitor of protein synthesis, 30 minutes before the training session in the object recognition (OR) test. Anisomycin administration prior to a 5 minute training protocol did not affect short term memory (STM) in *dream^−/−^* mice (discrimination indices [DI] = 0.3±0.032 and 0.27±0.099 for vehicle- and anisomycin-treated *dream^−/−^* mice, respectively: Fig. 2A), although the consolidation of OR memory was blocked (DI = 0.4±0.036 and 0.015±0.078 for vehicle- and anisomycin-treated *dream^−/−^* mice, respectively, *p*<0.001: Fig. 2B). Furthermore, anisomycin failed to alter the exploration times of *dream^−/−^* mice in the OR test (Table S1). Together, these results indicate that the enhanced learning observed in *dream^−/−^* mice is independent of protein synthesis

Finally, we investigated whether inhibition of the I_A_ current with 4-AP in wt mice affected learning and memory in the OR test (Fig. 2C and D). Administering 4-AP 30 minutes prior to the 5 minute training session increased OR STM (DI = 0.029±0.014 and 0.321±0.039 for vehicle- and 4-AP-treated wt mice, respectively, *p*<0.01, Fig. 2C) to similar level as in vehicle-injected mice trained for 15 minutes. By contrast, 4-AP-treated wt mice displayed poor consolidation of OR memory when the test was performed 24 hours after the initial 5 minute training session (DI = 0.045±0.055 and 0.169±0.016 for vehicle- and 4-AP-treated wt mice, respectively, *p*>0.05: Fig. 2D). Moreover, exploration times in the OR test did not differ significantly between vehicle and 4-AP-treated mice (Table S1), discarding a peripheral side effect of 4-AP. Together, these findings suggest that the decrease in the I_A_ facilitates the learning process in the OR test.

### Diminished I_A_ alters basal oscillatory hippocampal activity

**Figure 3:**
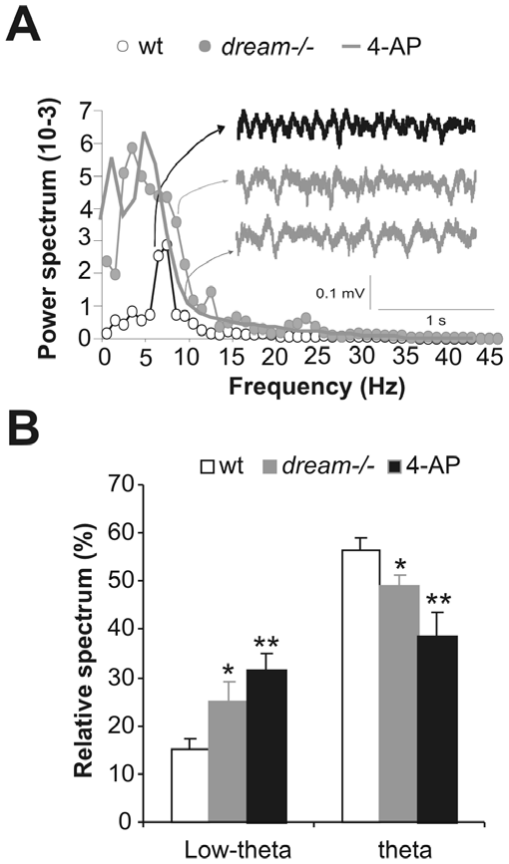
Kv4.2 channel blockade induces alterations in basal oscillatory hippocampal activity. (**A**) Power spectra of hippocampal local field activity recorded from the CA1 pyramidal layer of wt, *dream^−/−^* and 4-AP-treated wt mice during exploratory behavior. Two seconds of the basal electrocorticogram recordings are also shown. (**B**) Relative spectrum quantification (mean ± SEM) in the low theta and theta ranges for wt, *dream ^−/−^* and 4-AP-treated mice. n = 6 per group. * *p*≤0.05, ** *p*≤0.01.

The hippocampus is a cortical area involved in information processing and memory consolidation [35]. As the I_A_ is a determinant of neuronal excitability [32], we sought to determine the role of the I_A_ in hippocampal function. Mice were implanted with chronic stimulating and recording electrodes at hippocampal CA3-CA1 synapses, and recordings were obtained from freely moving mice. To determine the role of I_A_ in hippocampal function, we compared hippocampal electrical recordings from wt and *dream^−/−^* mice, as well as in vehicle- and 4-AP-treated wt mice. Basal hippocampal activity in *dream^−/−^* mice and in 4-AP-treated wt mice revealed differences in the amplitude of the electrocorticogram power spectrum with respect to vehicle-treated wt mice (Fig. 3A). The relative spectrum analysis revealed alterations in the theta (56.3±2.54%, 48.82±2.25% and 38.5±4.96% of the relative spectrum for wt, *dream^−/−^* and 4-AP-treated wt mice, respectively; F(21, 2) = 8.05, *p* = 0.001) and low-theta (15.02±2.19%, 25.05±4.45 and 31.19±3.94% of the relative spectrum for vehicle-treated wt, *dream^−/−^* and 4-AP-treated wt mice, respectively; F(21, 2) = 6.04, *p* = 0.004) amplitude bands during exploratory behavior (Fig. 3B). By contrast, there were no differences in basal synaptic activity, studied by paired pulse facilitation (PPF) with interpulse intervals from 50 to 200 ms, between vehicle-and 4-AP-treated wt mice (Fig. S1), nor between wt and *dream^−/−^* mice [30]. These findings indicate that I_A_ inhibition alters basal oscillatory hippocampal activity.

### Diminished I_A_ facilitates short-term hippocampal synaptic plasticity

**Figure 4:**
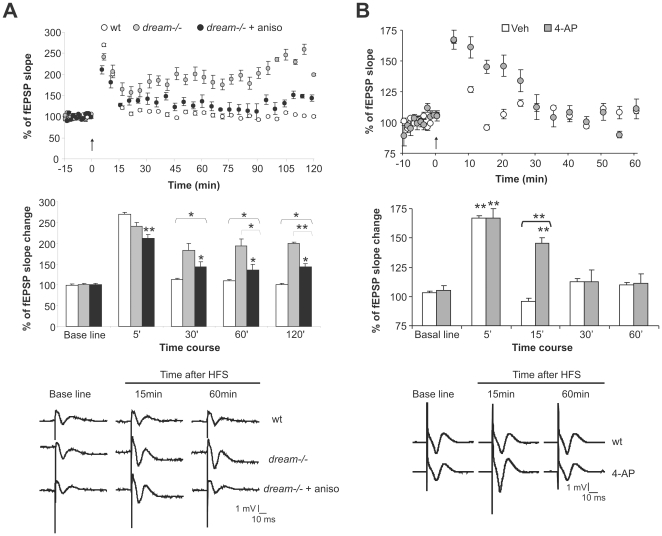
Reduced I_A_ decreases the stimulus threshold for short-term synaptic plasticity. (**A**) A single high-frequency stimulation (HFS, five trains, 200 Hz, 100 ms, at a rate of 1/s) evoked LTP at the CA3-CA1 synapse, lasting for up to 2 h in *dream^−/−^* mice (gray symbols). Anisomycin administration (white symbols) before HFS reduced the extension of LTP. A summary of the percent change in the fEPSP slope (mean ± SEM) and representative recordings at different times after a single HFS train in wt and *dream^−/−^* mice, in the presence or absence of anisomycin, is shown. (**B**) Effect of 4-AP administration on high-frequency stimulation (HFS) in wt mice. 4-AP administration evoked lasting short-term potentiation in wt mice. A summary of the changes in the fEPSP slope (mean ± SEM) and representative recordings at different times after a single HFS pulse in vehicle and 4-AP-treated mice is shown. n = 6 per group. *p≤0.05, ** *p*≤0.01.

We recently reported that *dream^−/−^* mice exhibit long-lasting LTP after a high frequency stimulation (HFS) protocol [30]. To determine whether inhibition of protein synthesis affected the facilitation of LTP in *dream^−/−^* CA3-CA1 synapses, we administered anisomycin to *dream^−/−^* mice 45 min before HFS delivery. Anisomycin administration reduced the late-LTP induced by one HFS train without affecting early-LTP (5 min after HFS: 241.29±7.7% and 211±10% following vehicle and anisomycin administration, respectively, *p* = 0.37. 30 min after HFS: 183.46±15.4% and 143±13% following vehicle and anisomycin administration, respectively, *p* = 0.096. 1 h after HFS: 194.03±1.54% and 136.37±14% following vehicle and anisomycin administration, respectively, *p* = 0.028. 2 h after HFS: 198.92±3% and 143±7.46% following vehicle and anisomycin administration, respectively, *p*<0.001: Fig. 4A). These data reveal the dissociation between early- and lasting-LTP facilitation after one HFS train with respect to new protein synthesis in *dream^−/−^* mice.

To determine the role of the I_A_ in short-term synaptic facilitation in *dream^−/−^* mice, we examined the effects of 4-AP on the HFS of the Schaffer's collateral-CA1 synapse in freely moving wt mice. This protocol only produced short-term changes in synaptic efficacy in wt mice lasting about 15 minutes (F(20, 4) = 13.16, *p*<0.001: Fig. 4B). However, the same pattern of stimulation produced a more sustained long-lasting synaptic enhancement, lasting about 30 minutes, when wt mice were administered with 4-AP (F(20, 4) = 13.21, *p*<0.001. 5 min after HFS: 167.18±7.87% and 166.78±2.28% in 4-AP-treated and vehicle-treated mice, respectively, *p*>0.05. 15 min after HFS: 145.22±5.2% and 96.12±2.35% in 4-AP-treated and vehicle-treated wt mice, *p*<0.01. 30 min after HFS: 112.65±19.06% and 112.27±3.2% in 4-AP-treated and vehicle-treated wt mice, *p*>0.05. 1 h after HFS: 111.12±8.13% and 109.63±2.4% in 4-AP-treated and vehicle-treated wt mice, *p*>0.05: Fig. 4B). Together these results suggest that the decrease in I_A_ is related to the facilitation of short-term changes in synaptic efficiency induced by a HFS protocol.

### The facilitation of learning induced by reduced IA is mediated by NR2B-containing NMDA receptors

**Figure 5:**
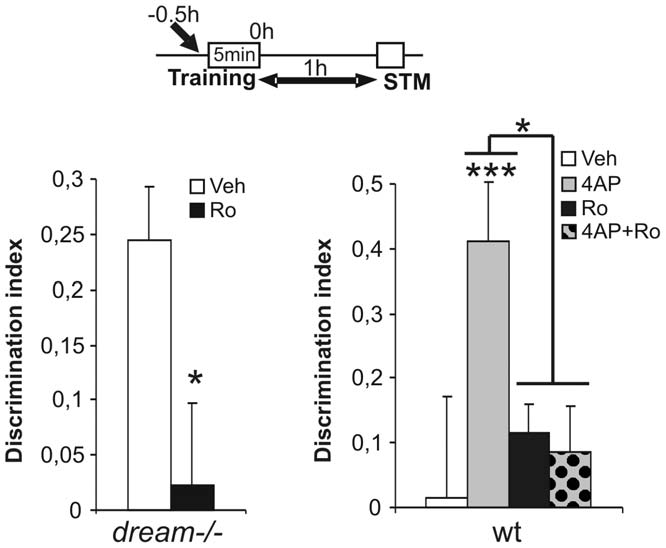
The facilitation of learning induced by decreased I_A_ is mediated by NR2B-containing NMDA receptors. The object recognition memory test was performed with a 5 min training session in *dream^−/−^* mice pretreated with Ro26-6981 or the vehicle alone (left graph, white and black bars, respectively), and in mice pretreated with 4-AP-treated in the presence or absence of Ro25-6981 (right graph). The discrimination indices during short-term memory (STM) are shown. n = at least 8 per group. * *p*≤0.05, *** *p*≤0.001.

Crosstalk between Kv4.2 channels, the primary mediators of the hippocampal I_A_, and the composition and activity of NMDARs has been described previously [27]. To determine whether NR2B-containing NMDARs play a role in the facilitation of learning when I_A_ is reduced, we performed the OR test using vehicle- or Ro25-6981-treated *dream^−/−^* mice. Ro25-6981 (5 mg/kg, s.c.) blocked STM in a 5 minute OR training protocol in *dream^−/−^* mice (DI = 0.24±0.047 and 0.02±0.074 in vehicle- and Ro25-6981-treated mice, respectively; F(30, 1) = 6.39, *p* = 0.016: Fig. 5). Finally, to assess the effects of inhibition of NR2B-containing NMDARs on the facilitated learning observed in 4-AP-treated wt mice, we co-administered 4-AP and Ro25-6981 30 minutes prior to the 5 minute OR training session. Accordingly, Ro25-6981 blocked the facilitation of learning induced by 4-AP (DI = 0.24±0.074 and 0.08±0.072 for 4-AP- and 4-AP+Ro25-6981-treated mice, respectively: t(22) = 4.22, *p* = 0.02, Fig. 5). Furthermore, all the drugs administered failed to alter the exploration times of *dream^−/−^* or wt mice in the OR test (Table S2). Together, all these data suggest that the facilitation of learning induced by decreased I_A_ is mediated by NR2B-containing NMDARs.

## Discussion

In neurons, the I_A_ plays important roles in processing dendritic signals, regulating action potential propagation, synaptic integration and the filtering of fast synaptic potentials [14], [32], [36]–[39], and modulating LTP [40]. Genetic manipulation of hippocampal Kv4.2, the main mediator of I_A_ [8], was recently shown to alter dendritic Ca^2+^ influx during back-propagation [8], [16], [17], [32]. This suggests that the regulation of back-propagation by I_A_ is important to control dendrite depolarization and the subsequent cellular cascades that influence the induction and/or maintenance of synaptic plasticity [41], [42]. However, to date the role of I_A_ in learning and memory remains unclear.

To address this issue, we used two models in which I_A_ was diminished: *dream^−/−^* mice and wild-type mice treated with the I_A_ inhibitor 4-AP. In both models, learning was facilitated in the object recognition test, using a 5 min training protocol. We recently showed that the DREAM protein modulates neuronal plasticity by determining the stimulation threshold required for long-term neuronal plasticity, thereby influencing learning consolidation and LTP [30], key events in learning and memory processes [43], [44]. Our results using *dream^−/−^* mice and the protein synthesis inhibitor anisomycin indicate that DREAM modulates both short- and long-term synaptic plasticity via distinct molecular pathways [30]. The differential localization-dependent functions of DREAM [18], [19] may explain its dual role in neuronal plasticity. Nuclear DREAM modulates long-lasting synaptic plasticity events and learning consolidation [30], [45] while cytoplasmic DREAM modulates short-term synaptic plasticity events and learning. Thus, while the role of DREAM in memory consolidation is related to its transcriptional activity [30], its role in short-term synaptic plasticity and learning may be due to compensatory effects that drive a decrease in I_A_ activity. This hypothesis is supported by the observed pharmacological inhibition of I_A_ by 4-AP. Indeed, some transgenic mouse models that also produce a small loss of IA [45], [46] have seen improvements in hippocampal-dependent learning tests. Contrary, the lack of kv4.2 channel although shows LTP enhancement, presents impairment in hippocampal-learning tests [47] It appears that LTP enhancement may have a more complicated relationship to learning: small gains in sensitivity lead to improvements while larger cellular changes may tip the balance toward learning impediment.

DREAM interacts with the Kv4 family of K^+^ channels, modulating their trafficking and permeability properties [19], [24], [31], [48]. Recent biochemical, molecular, genetic and electrophysiological data suggest that Kv channel interacting proteins (KChIPs) are critical for the formation of functional Kv4 channel complexes, and for the I_A_ [34], [49]. Our studies of Kv4.2 channel expression and activity in *dream^−/−^* mice revealed that the absence of DREAM decreases hippocampal Kv4.2 expression and I_A_ activity when compared to wt mice. These results are in agreement with those obtained with similar *dream^−/−^* mice [34], [50]. Moreover, kv4.2 deletion in mice alters the expression of both DREAM and KChIPs [8], [33], suggesting that the expression of Kv4.2 and DREAM (or KChIPs in general) is tightly coupled. It also suggests the existence of a feedback mechanism to prevent the accumulation of free KChIPs or Kv4.2. This could be achieved by coupling KChIP and Kv4.2 gene expression and/or through post-transcriptional/post-translational mechanisms. Importantly, the marked reductions in the expression of KChIP proteins in *kv 4.2 ^−/−^* mice are not evident at the transcriptional level, suggesting that post-translational mechanisms are responsible for the loss of KChIP proteins [34]. In fact, the co-expression of KChIPs and Kv4.2 leads to increased protein stability [48]. Together these findings suggest that the binding of KChIP and Kv4 proteins leads to mutual stabilization, increasing the levels of both the Kv4 subunit and accessory KChIP proteins.

The reduction or inhibition of I_A_ expression *in vivo* results in a decrease in the stimulus threshold required to induce lasting early-LTP with a single HFS, and a change in the basal electrical oscillatory pattern of the hippocampus (towards the low-theta oscillation range). At the cellular level, these alterations may be the consequence of increases in neuronal excitability, and in the dendritic back-propagation of action potential that occurs in the hippocampus in the absence of the I_A_ current [8], [48]. It is important to note that all these hippocampal events have previously been linked to the facilitation of learning and memory [30], [49]–[51]. In fact, our results show that decreased I_A_ activity facilitates learning, in contrast to situations in which these events facilitated both learning and memory. These findings suggest that decreased I_A_ is not sufficient to provoke the learning-induced changes in gene expression required for memory consolidation.

Hippocampal dendritic Kv4.2 channel surface expression is regulated by synaptic activity [17] and it decreases significantly during synaptic plasticity processes. This suggests a mechanism must exist to control synaptic integration through the regulation of the surface expression of the channels responsible for the I_A_. Synaptic plasticity processes are also influenced by the NR2 subunit type in NMDA receptors [49], [52]. In young rats LTP induces an immediate change in the NR2 subunit composition of synaptic NMDARs, favoring NR2A over NR2B. However, synaptic NR2B-containing receptors are still present in adult hippocampal synapses [53]–[55], suggesting that NR2B subunits are trafficked into the synapse. A relationship between functional I_A_ and the rapid and bidirectional remodeling of synaptic NMDAR subunit composition has been described [27]. Increased I_A_ induces a decrease in the contribution of the NR2B subunit to the total synaptic NMDAR current, while I_A_ knockdown increases the relative NR2B fraction. Thus, I_A_-dependent remodeling of synaptic NMDAR composition appears to be accompanied by changes in the ability to induce LTP. Here, we report that *in vivo* reduction of I_A_ facilitated the induction of early-LTP, similar to that produced by overexpression of a mutant Kv4.2 channel *in vitro* [27]. Whether this effect is due to increased activity of the NR2B-containing NMDARs is unknown, and will require further investigation. Nevertheless, the facilitated learning observed in mice following I_A_ reduction was blocked by inhibition of NR2B-containing NMDARs, suggesting that both learning and synaptic plasticity are regulated at molecular level by crosstalk between the I_A_ current and NR2B-containing NMDARs. Interestingly, recent studies described the modulation of NMDAR activity by DREAM [25], [26]. Taken together, these findings suggest that Kv4.2, DREAM and NMDAR proteins are integral components of a interacting complex that, via NMDAR activation, regulates the synaptic efficacy mediating synaptic plasticity and learning.

## Materials and Methods

### Transgenic mice

The *dream^−/−^* mice used in this study were those described previously [56]. All experimental protocols were also approved by the Ethics Committee of the Pablo de Olavide University (07/4-20/12/2008) in accordance with the European Community guidelines (86/609/EEC amended by Directive 2005/65/EC) and the Spanish regulations for the procurement and care of experimental animals (1201 RD/2005, October 10). Mice aged 4 to 6 months old were used for the behavioral experiments and the in vivo electrophysiological recordings.

### Drug administration

Mice were injected with 4-aminopyridine (4-AP, 1 mg/kg i.p., Tocris Cookson, Ballwing, MO) or the vehicle alone 30 minutes before behavioral or electrophysiological testing. This dose (1 mg/kg) of 4-AP was judged to be optimal from dose–response curves in the object recognition test and from electrophysiological recordings. A 5-folds dose (5 mg/kg) induced status epilepticus (revealed by electrophysiological recording) and did not facilitate cognition in cognition tests in healthy mice. *R*-(*R*,*S*)-α-(4-hydroxyphenyl)-β-methyl-4-(phenylmethyl)-1-piperidine propranol (Ro25-6981, Sigma-Aldrich, Madrid, Spain), a potent antagonist of the NR2B subunit, was dissolved in DMSO at a concentration of 5 mg/ml. Mice received subcutaneous injections of Ro25-6981 (5 mg/kg) or an equivalent volume of the vehicle alone. Anisomycin (Sigma, Madrid, Spain) was diluted in saline and dissolved in 1 N HCl. NaOH (1 N) was added to the solution until the pH was 7. Mice received subcutaneous injections of 25 mg/kg anisomycin or an equivalent volume of saline.

### Behavioral Tests

The object recognition memory test was performed as described by [49]. Briefly, mice were tested in a rectangular arena (55×40×40 cm) located in a room with dim lighting and constant background noise. In the object recognition protocol, two different objects were placed in the arena during the training phase. After a delay of 1 or 24 h, one object was changed to a novel object. The aim was to test the animal's memory of the original objects by measuring the amount of time spent exploring the novel object versus the familiar one. Selected objects consisted of plastic pieces with different forms and were thoroughly cleansed between trials to ensure the absence of olfactory cues. Before the experiment, mice were habituated to the arena in the absence of objects for 20 min each day over 2 days. On the day of testing, the mice were allowed to explore two objects for 5 min. to study learning and memory facilitation, or they were left for 15 min. Retention tests were performed either 1 or 24 h later by placing the mice back in the arena for a 10 min session and by randomly exchanging one of the familiar objects with a novel one. The time spent exploring each object was recorded, and the relative exploration of the novel object was expressed by a discrimination index [DI = (*t*_novel_−*t*_familiar_)/(*t*_novel_+*t*_familiar_)]. The criteria for exploration were based strictly on active exploration, during which the mouse had both forelimbs within a circle of 1.5 cm around the object, with its head oriented toward it, or was touching it with its vibrissae.

### Electrophysiology

Electrophysiology experiments were performed as described previously [49]. Briefly, bipolar stimulating electrodes were implanted on the Schaffer's collateral-commissural pathway of the dorsal hippocampus (from Bregma, AP: 1.5; L: 2.2 mm; depth from brain surface, 1.0–1.5 mm), and two recording electrodes were implanted in the ipsilateral stratum radiatum, underneath the CA1 area (from Bregma, AP: 2.2; L: 2.2 mm; depth from brain surface, 1.0–1.5 mm). All in-vivo recordings were performed at least 7 days after surgery. To evoke LTPs, each animal received five pulse trains (200 Hz, 100 ms) at a rate of 1/s. This protocol was administered either once or a total of six times at intervals of 1 min. The hippocampal activity recorded was stored digitally on a computer through an analog/digital converter (CED 1401 Plus, Cambridge, England) at a sampling frequency of 11–22 kHz and with an amplitude resolution of 12 bits. Computer programs (Spike 2 and SIGAVG from CED) were adapted to represent the extracellular synaptic field potential (fEPSP) recordings, and the slope of the evoked fEPSPs was collected as the first derivative (i.e., V/s) of the fEPSP records (V). Accordingly, five successive evoked field synaptic potentials at intervals of 5 min were averaged, and the mean value of the slope was determined for the rise-time period (i.e., the period of the slope between the initial 10% and the final 10% of the evoked field potential).

The power spectrum of the hippocampal field activity was calculated using the fast Fourier transformation with a Hanning window. This parameter was expressed as the relative power and averaged across each session. The average was analyzed and compared using the wide-band model, considering the following bands: low theta (2–4 Hz) and theta (4–9 Hz).

For paired-pulse facilitation, two stimuli of an intensity that evoked 35–40% of the maximum fEPSP response were delivered with an inter-stimulus interval of 50–200 ms. The percentage facilitation was calculated as (slope S2/slope S1) ×100.

### Whole cell recordings in acute hippocampal slices

Mice (15–20 days old) were anesthetized by hypothermia, decapitated and their brain hemispheres were quickly dissected in an ice-cold oxygenated (95% O_2_, 5% CO_2_) sucrose artificial cerebrospinal fluid (S-aCSF, in 26 mM NaHCO_3_, 10 mM glucose, 3 mM KCl, 1.25 mM NaH_2_PO_4_, 2 mM MgCl_2_ and 218 mM sucrose). Transverse hippocampal slices (300–400 µm thick) were obtained on a vibroslicer (World Precision Instruments) and they were transferred to normal oxygenated aCSF (without sucrose but with 130 mM NaCl, 2 mM CaCl_2_ added) and incubated at 36°C for at least 30 min before recording.

Individual slices were placed in the recording chamber and perfused continuously (∼3 ml/min) with oxygenated aCSF at 31°C. Patch pipettes (2–6 MΩ) were pulled from borosilicate glass and filled with an internal solution containing 17.5 mM KCl, 122.5 mM KGluconate, 9 mM NaCl; 1 mM MgCl_2_; 10 mM HEPES, 0.2 mM EGTA, 3 mM Mg-ATP and 0.3 mM GTP-Tris (pH 7.2). Whole cell voltage clamp recordings were obtained from the soma of CA1 pyramidal neurons using infrared differential interference contrast optics. The access resistance was <20 MΩ and the results were discarded when changes of more than 20% were observed. Currents were recorded using a Multiclamp 700B amplifier (Molecular Devices), low-pass bessel filtered at 5 kHz and digitalized at 10 kHz using a computer equipped with a Digidata 1322A data acquisition board and pCLAMP9.2 software (both from Molecular Devices). Series resistance was routinely compensated for by 65–75%.

To isolate whole-cell voltage-dependent K^+^ currents in CA1 pyramidal neurons, the aCSF was replaced with a modified extracellular solution containing nominally zero Ca^2+^, 200 µM CdCl_2_ and 1 µM tetrodotoxin (TTX) to block Ca^2+^ currents, Ca^2+^-activated K^+^ currents and fast Na^+^ currents. The leak and capacitive currents were digitally subtracted online by applying a P/4 subtraction protocol. To separate the IA from the total outward potassium current (I_Total_) we used a standard two-step voltage protocol consisting of a 300 ms hyperpolarizing prepulse at −90 mV followed by two sequential depolarizing steps to +40 mV, each 200 ms in duration, and separated by a 1 s interval at −40 mV. The I_A_ was activated by depolarization from −90 mV but not from a holding potential of −40 mV. Thus, the I_A_ was obtained by digitally subtracting the outward current response elicited by the second depolarizing pulse from that generated by the first depolarization. The data were analyzed off-line using pClamp 9.2 software, and the amplitudes of the I_A_ and delayed rectifier K^+^ current (I_KDR_) components were measured as the peak of the subtracted response and the steady-state current at the end of the second depolarizing pulse step, respectively. The current density was calculated by dividing the current amplitude by the cell capacitance.

### Western blotting

Immunoblotting was performed as previously described elsewhere [20], [57] and three mice were used for each group. The antibodies used were raised against Kv4.2 (sc-11680, Santa Cruz, 1∶500) and actin (sc-1615, Santa Cruz, 1∶1000).

### Analysis of mRNA expression by reverse transcription-PCR

Total RNA from brain tissue was extracted using the Tripure reagent (Roche Products, Hertfordshire, UK). The RNA from at least six animals per group was used for reverse transcription (RT)-PCR experiments using the kv4.2 PCR primers: 5′-ATCGCCCATCAAGTCACAGTC-3′ and 5′-CCGACACATTGGCATTAGGAA-3′. Arbitrary units were calculated as the ratio of the optical density band of the gene studied in the 25–30^th^ cycle to that of the *gadph* housekeeping gene (glyceraldehyde 3-phosphate dehydrogenase) in the 15th cycle of amplification. One unit was considered as the ratio corresponding to the band with the lowest optical density of the gene studied in each experiment. Three mice per groups were used and the PCR reactions were performed in triplicate.

### Statistical analysis

Statistical analyses were performed using the SPSS package for Windows (SPSS, Chicago, IL). Unless otherwise indicated, the data are represented as the mean ± SEM. The data were analyzed using a two-way ANOVA, with time or session as the repeated measure, and coupled to a contrast analysis where required. One-way ANOVA was used to assess the statistical differences between groups.

## Supporting Information

Figure S1 **4-AP does not affect hippocampal basal glutamatergic transmission.** Basal excitatory neurotransmission was measured using paired-pulse facilitation with interpulse intervals from 50 to 200 ms in the presence or absence of 4-AP. Lines represent the percentage of paired-pulse facilitation as a function of interpulse interval in vehicle- and 4-AP-treated mice (*n* = 6 per group).
(TIF)

Table S1 Total object exploration times (in seconds) of wt or *dream^−/−^* mice treated with vehicle or the drug indicated 15 min before the 5-minute OR memory training session. STM, short-term memory; LTM, long-term memory.
(DOC)

Table S2 Total object exploration times (in seconds) of wt or *dream^−/−^* mice treated with vehicle or the drug indicated 15 min before the 5-minute OR memory training session. STM, short-term memory.
(DOC)

## References

[1] Kandel ER (2001). The molecular biology of memory storage: a dialogue between genes and synapses.. Science.

[2] Dudai Y (2004). The neurobiology of consolidations, or, how stable is the engram?. Annu Rev Psychol.

[3] Kelleher RJ, Govindarajan A, Tonegawa S (2004). Translational regulatory mechanisms in persistent forms of synaptic plasticity.. Neuron.

[4] Klann E, Dever TE (2004). Biochemical mechanisms for translational regulation in synaptic plasticity.. Nat Rev Neurosci.

[5] Inda MC, Delgado-García JM, Carrión AM (2005). Acquisition, consolidation, reconsolidation, and extinction of eyelid conditioning response require de novo protein synthesis.. J Neurosci.

[6] Sutton MA, Schuman EM (2006). Dendritic protein synthesis, synaptic plasticity, and memory.. Cell.

[7] Romero-Granados R, Fontán-Lozano A, Delgado-García JM, Carrión AM (2010). From learning to forgetting: behavioral, circuitry, and molecular properties define the different functional states of the recognition memory trace.. Hippocampus.

[8] Chen X, Yuan LL, Zhao C, Birnbaum SG, Frick A (2006). Deletion of Kv4.2 gene eliminates dendritic A-type K+ current and enhances induction of long-term potentiation in hippocampal CA1 pyramidal neurons.. J Neurosci.

[9] Johnston D, Hoffman DA, Magee JC, Poolos NP, Watanabe S (2000). Dendritic potassium channels in hippocampal pyramidal neurons.. J Physiol (Lond).

[10] Johnston D, Christie BR, Frick A, Gray R, Hoffman DA (2003). Active dendrites, potassium channels and synaptic plasticity.. Philos Trans R Soc Lond B Biol Sci.

[11] Storm JF (2000). K(+) channels and their distribution in large cortical pyramidal neurones.. J Physiol.

[12] Sheng M, Tsaur ML, Jan YN, Jan LY (1992). Subcellular segregation of two A-type K+ channel proteins in rat central neurons.. Neuron.

[13] Maletic-Savatic M, Lenn NJ, Trimmer JS (1995). Differential spatiotemporal expression of K+ channel polypeptides in rat hippocampal neurons developing in situ and in vitro.. J Neurosci.

[14] Ramakers GM, Storm JF (2002). A postsynaptic transient K(+) current modulated by arachidonic acid regulates synaptic integration and threshold for LTP induction in hippocampal pyramidal cells.. Proc Natl Acad Sci USA.

[15] Watanabe S, Hoffman DA, Migliore M, Johnston D (2002). Dendritic K+ channels contribute to spike-timing dependent long-term potentiation in hippocampal pyramidal neurons.. Proc Natl Acad Sci USA.

[16] Frick A, Magee J, Johnston D (2004). LTP is accompanied by an enhanced local excitability of pyramidal neuron dendrites.. Nat Neurosci.

[17] Kim J, Jung SC, Clemens CM, Petralia RS, Hoffman DA (2007). Regulation of dendritic excitability by activity-dependent trafficking of the A-type K+ channel subunit Kv4.2 in hippocampal neurons.. Neuron.

[18] Carrion AM, Link WA, Ledo F, Mellström B, Naranjo JR (1999). DREAM is a Ca^2+^-regulated transcriptional repressor.. Nature.

[19] An WF, Bowlby MR, Betty M, Cao J, Ling HP (2000). Modulation of A-type potassium channels by a family of calcium sensors.. Nature.

[20] Campos D, Jímenez-Díaz L, Carrión AM (2003). Ca(2+)-dependent prodynorphin transcriptional derepression in neuroblastoma cells is exerted through DREAM protein activity in a kinase-independent manner.. Mol Cell Neurosci.

[21] Ledo F, Carrión AM, Link WA, Mellström B, Naranjo JR (2000). DREAM-alphaCREM interaction via leucine-charged domains derepresses downstream regulatory element-dependent transcription.. Mol Cell Biol.

[22] Ledo F, Mellström B, Kremer L, Naranjo JR (2002). Ca2+-dependent block of CREB-CBP transcription by repressor DREAM.. EMBO J.

[23] Rivas M, Mellstrom B, Naranjo JR, Santisteban P (2004). Transcriptional repressor DREAM interacts with thyroid transcription factor-1 and regulates thyroglobulin gene expression.. J Biol Chem.

[24] Takimoto K, Yang EK, Conforti L (2002). Palmitoylation of KChIP splicing variants is required for efficient cell surface expression of Kv4.3 channels.. J Biol Chem.

[25] Wu LJ, Mellström B, Wang H, Ren M, Domingo S (2010). DREAM (Downstream Regulatory Element Antagonist Modulator) contributes to synaptic depression and contextual fear memory.. Mol Brain.

[26] Zhang Y, Su P, Liang P, Liu T, Liu X (2010). The DREAM protein negatively regulates the NMDA receptor through interaction with the NR1 subunit.. J Neurosci.

[27] Jung SC, Kim J, Hoffman DA (2008). Rapid, Bidirectional Remodeling of Synaptic NMDA Receptor Subunit Composition by A-type K+ Channel Activity in Hippocampal CA1 Pyramidal Neurons.. Neuron.

[28] Jung SC, Eun SY, Kim J, Hoffman DA (2011). Kv4.2 block of long-term potentiation is partially dependent on synaptic NMDA receptor remodeling.. Brain Res Bull.

[29] Lei Z, Deng P, Li Y, Xu ZC (2010). Downregulation of Kv4.2 channels mediated by NR2B-containing NMDA receptors in cultured hippocampal neurons.. Neuroscience.

[30] Fontán-Lozano A, Romero-Granados R, del-Pozo-Martín Y, Suárez-Pereira I, Delgado-García JM (2009). Lack of DREAM protein enhances learning and memory and slows brain aging.. Curr Biol.

[31] Ruiz-Gomez A, Mellström B, Tornero D, Morato E, Savignac M (2007). G Protein-coupled Receptor Kinase 2-mediated Phosphorylation of Downstream Regulatory Element Antagonist Modulator Regulates Membrane Trafficking of Kv4.2 Potassium Channel.. J Biol Chem.

[32] Kim J, Wei DS, Hoffman DA (2005). Kv4 potassium channel subunits control action potential repolarization and frequency-dependent broadening in rat hippocampal CA1 pyramidal neurones.. J Physiol.

[33] Menegola M, Trimmer JS (2006). Unanticipated region- and cell-specific downregulation of individual KChIP auxiliary subunit isotypes in Kv4.2 knock-out mouse brain.. J Neurosci.

[34] Norris AJ, Foeger NC, Nerbonne JM (2010). Interdependent Roles for Accessory KChIP2, KChIP3, and KChIP4 Subunits in the Generation of Kv4-Encoded IA Channels in Cortical Pyramidal Neurons.. J Neurosci.

[35] Squire LR, Zola-Morgan S (1991). The medial temporal lobe memory system.. Science.

[36] Cash S, Yuste R (1998). Input summation by cultured pyramidal neurons is linear and position-independent.. J Neurosci.

[37] Goldberg JH, Tamas G, Yuste R (2003). Ca2+ imaging of mouse neocortical interneurone dendrites: Ia-type K+ channels control action potential backpropagation.. J Physiol.

[38] Hoffman DA, Magee JC, Colbert CM, Johnston D (1997). K+ channel regulation of signal propagation in dendrites of hippocampal pyramidal neurons.. Nature.

[39] Schoppa NE, Westbrook GL (1999). Regulation of synaptic timing in the olfactory bulb by an A-type potassium current.. Nat Neurosci.

[40] Kim J, Hoffman DA (2008). Potassium channels: newly found players in synaptic plasticity.. Neuroscientist.

[41] Bliss TV, Collingridge GL (1993). A synaptic model of memory: long-term potentiation in the hippocampus.. Nature.

[42] Malenka RC, Nicoll RA (1999). Long-term potentiation - a decade of progress?. Science.

[43] Daoudal G, Debanne D (2003). Long-term plasticity of intrinsic excitability: learning rules and mechanisms.. Learn Mem.

[44] Gruart A, Muñoz MD, Delgado-García JM (2006). Involvement of the CA3-CA1 synapse in the acquisition of associative learning in behaving mice.. J Neurosci.

[45] Alexander JC, McDermott CM, Tunur T, Rands V, Stelly C (2009). The role of calsenilin/DREAM/KChIP3 in contextual fear conditioning.. Learn Mem.

[46] Lilliehook C, Bozdagi O, Yao J, Gomez-Ramirez M, Zaidi NF (2003). Altered Abeta formation and long-term potentiation in a calsenilin knock-out.. J Neurosci.

[47] Lockridge A, Yuan L-L (2010). Spatial learning deficits in mice lacking A-type K^+^ channel subunits.. Hippocampus.

[48] Shibata R, Misonou H, Campomanes CR, Anderson AE, Schrader LA (2003). A fundamental role for KChIPs in determining the molecular properties and trafficking of Kv4.2 potassium channels.. J Biol Chem.

[49] Fontán-Lozano A, Sáez-Cassanelli JL, Inda MC, de los Santos-Arteaga M, Sierra-Domínguez SA (2007). Caloric restriction increases learning consolidation and facilitates synaptic plasticity through mechanisms dependent on NR2B subunits of the NMDA receptor.. J Neurosci.

[50] Rhodes KJ, Carroll KI, Sung MA, Doliveira LC, Monaghan MM (2004). KChIPs and Kv4 subunits as integral components of A-type potassium channels in mammalian brain.. J Neurosci.

[51] Fontán-Lozano A, Suárez-Pereira I, Delgado-García JM, Carrión AM (2011). The M-current inhibitor XE991 decreases the stimulation threshold for long-term synaptic plasticity in healthy mice and in models of cognitive disease.. Hippocampus.

[52] Bellone C, Nicoll RA (2007). Rapid bidirectional switching of synaptic NMDA receptors.. Neuron.

[53] Al-Hallaq RA, Conrads TP, Veenstra TD, Wenthold RJ (2007). NMDA di-heteromeric receptor populations and associated proteins in rat hippocampus.. J Neurosci.

[54] Kohr G, Jensen V, Koester HJ, Mihaljevic AL, Utvik JK (2003). Intracellular domains of NMDA receptor subtypes are determinants for long-term potentiation induction.. J Neurosci.

[55] Thomas CG, Miller AJ, Westbrook GL (2006). Synaptic and extrasynaptic NMDA receptor NR2 subunits in cultured hippocampal neurons.. J Neurophysiol.

[56] Cheng HY, Pitcher GM, Laviolette SR, Whishaw IQ, Tong KI (2002). DREAM is a critical transcriptional repressor for pain modulation.. Cell.

[57] de los Santos-Arteaga M, Sierra-Domínguez SA, Fontanella GH, Delgado-García JM, Carrión AM (2003). Analgesia induced by dietary restriction is mediated by the kappa-opioid system.. J Neurosci.

