# Supplementary figures and images for: The A-Current Modulates Learning via NMDA Receptors Containing the NR2B Subunit
Source: PLoS One. 2011 Sep 26;6(9):e24915. doi: 10.1371/journal.pone.0024915 (PMC3180285; doi:10.1371/journal.pone.0024915)

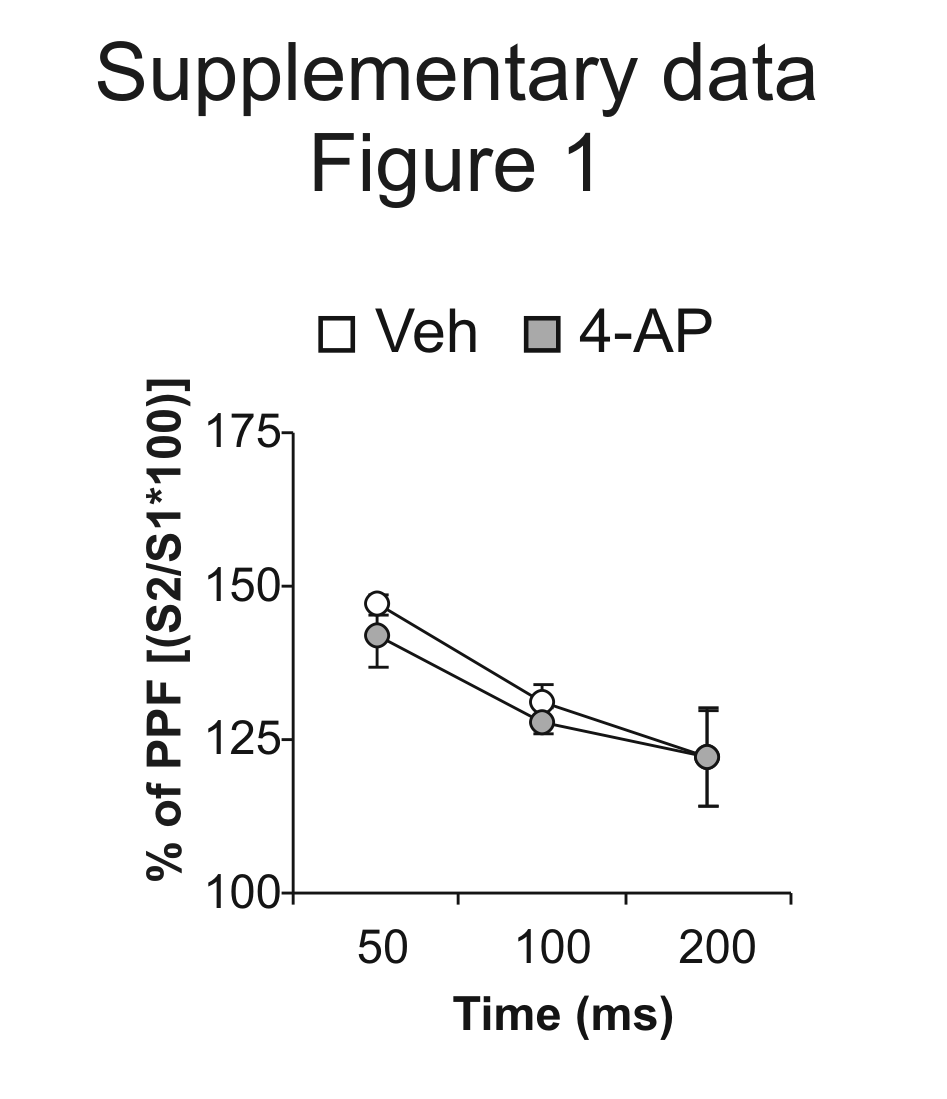

Supplement: Figure S1 — 4-AP does not affect hippocampal basal glutamatergic transmission. Basal excitatory neurotransmission was measured using paired-pulse facilitation with interpulse intervals from 50 to 200 ms in the presence or absence of 4-AP. Lines represent the percentage of paired-pulse facilitation as a function of interpulse interval in vehicle- and 4-AP-treated mice (n = 6 per group). (TIF) [file pone.0024915.s001.tif]
