# Supplementary material for: The A-Current Modulates Learning via NMDA Receptors Containing the NR2B Subunit
Source: PLoS One. 2011 Sep 26;6(9):e24915. doi: 10.1371/journal.pone.0024915 (PMC3180285; doi:10.1371/journal.pone.0024915)
Supplement: Table S1 — Total object exploration times (in seconds) of wt or dream−/− mice treated with vehicle or the drug indicated 15 min before the 5-minute OR memory training session. STM, short-term memory; LTM, long-term memory. (DOC) [file pone.0024915.s002.doc]

**Table 1.** Total object exploration times (in seconds) of wt or *dream^-/-^* mice treated with vehicle or the drug indicated 15min before the 5-minute OR memory training session.

STM, short-term memory; LTM, long-term memory

| OR memory: exploration time (s) per session in 5 min training protocol | | | |
| --- | --- | --- | --- |
|  | Training | STM | LTM |
| wt | 20.12 ± 2.22 | 31.12 ± 5.42 | 30.00 ± 3.07 |
| wt + 4-AP | 22.52 ± 4.15 | 36.21 ± 3.93 | 34.15 ± 4.48 |
|  |  |  |  |
| OR memory: exploration time (s) per session in 5 min training protocol | | | |
|  | Training | STM | LTM |
| wt | 28.60 ± 4.67 | 45.50 ± 2.90 | 40.60 ± 5.60 |
| *dream^-/-^* | 19.60 ±3.69 | 33.60 ± 3.17 | 29.25 ± 2.28 |
| *dream^-/-^* + aniso | 17.4 ± 6.04 | 25.2 ± 7.01 | 46.00 ± 5.33 |
