# Supplementary material for: The A-Current Modulates Learning via NMDA Receptors Containing the NR2B Subunit
Source: PLoS One. 2011 Sep 26;6(9):e24915. doi: 10.1371/journal.pone.0024915 (PMC3180285; doi:10.1371/journal.pone.0024915)
Supplement: Table S2 — Total object exploration times (in seconds) of wt or dream−/− mice treated with vehicle or the drug indicated 15 min before the 5-minute OR memory training session. STM, short-term memory. (DOC) [file pone.0024915.s003.doc]

**Table 2.** Total object exploration times (in seconds) of wt or *dream^-/-^* mice treated with vehicle or the drug indicated 15min before the 5-minute OR memory training session. STM, short-term memory.

| OR memory: exploration time (s) per session in 5 min training protocol | | |
| --- | --- | --- |
|  | Training | STM |
| *dream^-/-^* + saline | 16.5 ± 0.95 | 24.6 ± 1.12 |
| *dream^-/-^* + Ro25-6981 | 18.3 ± 1.43 | 26.3 ± 1.73 |
|  |  |  |
| OR memory: exploration time (s) per session in 5 min training protocol | | |
|  | Training | STM |
| wt | 21 ± 2 | 22 ± 3.82 |
| wt + 4-AP | 20.5 ± 2.32 | 21.66 ± 1.52 |
| wt + Ro25-6981 | 24.5 ± 2.83 | 27.3 ± 4.31 |
| wt + 4-AP + Ro25-6981 | 20.66 ± 1.94 | 20.83 ± 1.51 |
